# Geographical Variation in Antibiotic-Resistant *Escherichia coli* Isolates from Stool, Cow-Dung and Drinking Water

**DOI:** 10.3390/ijerph9030746

**Published:** 2012-03-02

**Authors:** Krushna Chandra Sahoo, Ashok J. Tamhankar, Soumyakanta Sahoo, Priyadarshi Soumyaranjan Sahu, Senia Rosales Klintz, Cecilia Stålsby Lundborg

**Affiliations:** 1 Division of Global Health (IHCAR), Department of Public Health Sciences, Karolinska Institutet, Nobels väg 9, SE 171 77 Stockholm, Sweden; Email: senia.rosales.1@ki.se (S.R.K.); cecilia.stalsby.lundborg@ki.se (C.S.L.); 2 Indian Initiative for Management of Antibiotic Resistance (IIMAR), Department of Environmental Medicine, R.D. Gardi Medical College, Ujjain 456 006, India; Email: ejetee@gmail.com; 3 Department of Microbiology, Super Religare Laboratories Limited, Kalinga Hospital, Bhubaneswar 751 023, India; Email: sk.soumya@gmail.com; 4 Department of Microbiology, Kalinga Institute of Medical Sciences and School of Biotechnology, KIIT University, Bhubaneswar 751 024, India; Email: priyadarshi_sahu@yahoo.com

**Keywords:** antibiotic resistance, non-coastal, coastal, ciprofloxacin resistance, Odisha, India

## Abstract

Little information is available on relationships between the biophysical environment and antibiotic resistance. This study was conducted to investigate the antibiotic resistance pattern of *Escherichia coli* isolated from child stool samples, cow-dung and drinking water from the non-coastal (230 households) and coastal (187 households) regions of Odisha, India. Susceptibility testing of *E. coli* isolates (*n* = 696) to the following antibiotics: tetracycline, ampicillin/sulbactam, cefuroxime, cefotaxime, cefixime, cotrimoxazole, amikacin, ciprofloxacin, norfloxacin and nalidixic acid was performed by the disk diffusion method. Ciprofloxacin minimum inhibitory concentration (MIC) values were determined for ciprofloxacin-resistant isolates (*n* = 83). Resistance to at least one antibiotic was detected in 90% or more of the *E. coli* isolates. Ciprofloxacin MIC values ranged from 8 to 32 µg/mL. The odds ratio (OR) of resistance in *E. coli* isolates from children’s stool (OR = 3.1, 95% CI 1.18–8.01), cow-dung (OR = 3.6, 95% CI 1.59–8.03, *P* = 0.002) and drinking water (OR = 3.8, 95% CI 1.00–14.44, *P* = 0.049) were higher in non-coastal compared to coastal region. Similarly, the co-resistance in cow-dung (OR = 2.5, 95% CI 1.39–4.37, *P* = 0.002) and drinking water (OR = 3.2, 95% CI 1.36–7.41, *P* = 0.008) as well as the multi-resistance in cow-dung (OR = 2.2, 95% CI 1.12–4.34, *P* = 0.022) and drinking water (OR = 2.7, 95% CI 1.06–7.07, *P* = 0.036) were also higher in the non-coastal compared to the coastal region.

## 1. Introduction

The association of environmental factors with antibiotic resistance is an emerging issue [1,2]. The components of the environment are natural, physical as well as social and behavioural [3] and they all play an important role in modifying community health and effectiveness of medicines. A number of studies document the effect of social and behavioural factors on antibiotic resistance [4], while little information is available on relationships between the biophysical environment and antibiotic resistance. 

*Escherichia coli* is present in the intestinal tracts of both humans and animals, is released into the environment through faecal material and is therefore used as an indicator of faecal contamination [5]. *E. coli* is also a reservoir for antibiotic resistance genes [6]. In the natural environment the resistant bacteria and resistance genes from animal or environmental origin might transfer to humans [7,8]. Use of antibiotics is one of the factors contributing to resistance [4,9]. Among the antibiotics commonly used both in human and veterinary medicine are the fluoroquinolones [10], with ciprofloxacin being the most consumed fluoroquinolone worldwide [9]. As a consequence of this, bacterial fluoroquinolone resistance has been reported both in both humans and animals [11]. 

In the state of Odisha, endowed with multiple environmental niches, the infectious disease burden is high in comparison to other Indian states [12], however, there is a lack of healthcare facilities. Furthermore, there is a lack of studies on antibiotic resistance. Globally, there is also a lack of studies on prevalence of antibiotic resistance in relation to different geographical regions. Therefore, we decided to investigate the antibiotic resistance patterns in *E. coli* isolates from two contrasting biophysical environments—the coastal and non-coastal regions of Odisha. Children’s stools, cow-dung and drinking water samples were included in this study as representatives of both community and environmental sources. 

## 2. Experimental Section

### 2.1. Study Settings

This cross sectional study was conducted in two districts—“Malkangiri” (non-coastal) and “Puri” (coastal) of the state of Odisha (total population of 41.9 million) in India. The distinctive features of these two districts are given in Table 1 [13,14]. 

**Table 1:** Environmental variables of the study setting, Malkangiri and Puri, Odisha.

Geographical regions and environmental variables	Malkangiri (Non-coastal)	Puri (Coastal)
Physical components		
Temperature (Maximum, Minimum)	47 °C, 11 °C	36 °C, 13 °C
Average yearly rainfall in mm	1465	1586
Natural components		
Height from the sea level in metres	196	6
Forest cover in percentage	38	3
Social components		
Total population in million	0.61	1.69
Population Density per Sq. Km	106	488
Literacy Rate in percentage	49	85

### 2.2. Sampling and Data Collection

The sample size was calculated by considering the differences in proportions of antibiotic resistance between the two geographical regions (non-coastal and coastal) for each type of sample (children’s stools, cow-dung and drinking water), attempting to test the null hypothesis of no difference between proportions, with a conservative estimated proportion of antibiotic resistance equal to 50% in each comparison group; alpha level = 0.05, power = 0.80 and minimum difference in proportions considered significant equal to 20%. The required sample size obtained by this calculation was at least 103 isolates from each comparison group. 

Criteria for the selection of the actual sampling site were distance from the seacoast, vehicular accessibility and feasibility of transportation of samples to the microbiology laboratory. The census block (administrative area) of “Kalimela”, located at a distance of about 200 km from the sea coast, in the hilly district of “Malkangiri”, satisfied these criteria and was taken as a sampling site representing the non-coastal region. According to Integrated Coastal Zone Management (ICZM) ten kilometers of the landside of coastal structures is considered as a coastal area [15]. The census block of “Brahmgiri” in the district of “Puri”, offered an opportunity of sampling in an area closer to the sea coast (less than 6 km from seacoast) was selected as a sampling site representing the coastal area. Villages were selected randomly from each census block. Within each village, all households having at least one cow and at least one child of three to nine years of age in good state of health (as reported by the head of the family) were selected for sampling. Children and cows are both commonly present in both areas. Children’s stool and cow-dung samples were thus chosen as they reflect the respective communities. In each selected household samples collected were from: child stool (from one child three to nine years of age), cow-dung (from one cow) and drinking water (500 mL from the drinking water storage container). The samples were collected in sterile containers in a single sampling time in the morning. The collection of samples from a village was continued until samples were collected from all the eligible households. When no more suitable households were left in a village, sampling was continued in the next geographically contiguous village. The process of sampling was continued until the required sample size was achieved in the coastal and non-coastal areas respectively. All samples were transported in an icebox (temperature less than 10 °C), to the microbiology laboratory for analysis. 

During the sample collection process, along with the samples, information on the age and sex of the child, antibiotic treatment history of both the child and the cow, socioeconomic status of the family [16], source of drinking water, and defecation practices of household’s members was collected. 

Before sample collection, the procedures and purpose of the study were explained in the local language to the heads of the households and to the children. Informed consent was obtained from the heads of the households. The ethical committee of the Kalinga Institute of Medical Sciences, Odisha, approved the study. The samples were collected from April 2010 to May 2011. A pilot study was conducted before the main study to assess the feasibility. 

### 2.3. Isolation and Identification of *E. coli*

The bacterial culture and isolation were carried out within 6 to 48 h after sampling following standard operating procedure as described by Clinical Laboratory Standards Institute (CLSI) guidelines [17]. For each child’s stool and cow-dung sample, a faecal suspension was prepared by adding a portion of the specimen onto 2–3 mL of 0.1% peptone water to make it 1:10 dilution. One loop full of faecal suspension was streaked onto MacConkey agar and was incubated at 37 °C for 24 h. The lactose fermenting colonies were initially assessed by their characteristic growth on MacConkey agar. Each isolate was further confirmed by standard biochemical tests designated for *E. coli* (indole, methyl red, Voges-Proskauer, and citrate utilization test) [17]. The enumeration of *E. coli* from drinking water was performed by a two membrane filtration technique using membrane lauryl sulphate agar incubated at 37 °C and 44 °C [18]. 

### 2.4. Antibiotic Susceptibility Testing

One *E. coli* isolate per sample was tested for susceptibility by the Kirby-Bauer’s disk diffusion method against the following antibiotic groups: tetracyclines (tetracycline 30 µg), penicillins (ampicillin/sulbactam 10 µg/10 µg), 2nd generation cephalosporins (cefuroxime 30 µg), 3rd generation cephalosporins (cefotaxime 30 µg and cefixime 5 µg), cotrimoxazole (1.25 µg/23.75 µg), aminoglycosides (amikacin 30 µg) and quinolones (ciprofloxacin 5 µg, norfloxacin 10 µg and nalidixic acid 30 µg) [17]. This panel of antibiotics was selected based on the antibiotic prescription patterns in local hospitals and veterinary dispensaries, and the CLSI guidelines [17]. Overnight cultures, grown on trypticase soy broth (optical density adjusted to MacFarland 0.5), were spread evenly on Mueller-Hinton agar plates. The plates were incubated at 37 °C for 24 h. The zones of inhibition were measured and interpreted as resistant or sensitive according to CLSI guidelines [17]. 

Co-resistance (resistance to two antibiotic groups) and multi-resistance (resistance to at least three different antibiotic groups) were recorded. 

### 2.5. Determination of Ciprofloxacin Minimum Inhibitory Concentration

Minimum inhibitory concentration (MIC) values for ciprofloxacin were determined by an Epsilometer test for a sub-set of ciprofloxacin-resistant isolates (*n* = 83). Only isolates that showed resistance to ciprofloxacin (irrespective of resistance to other antibiotics or not) were subjected to MIC. The first 35% isolates from each sample type, from each district were serially selected. Commercially available ciprofloxacin EzyMIC^TM^ Strips (HiMedia, Mumbai, India) with a MIC range of 0.002 to 32 µg were used following manufacturer’s instructions. *E. coli* ATCC 25922 was used as control strain in both Kirby-Bauer disk diffusion and epsilometer tests.

### 2.6. Statistical Analysis

The data were entered in Excel version Office 2007 and then transferred to Stata 10.1 (Stata Corp. College Station, TX, USA) software for statistical analysis. Differences in prevalence of antibiotic resistance, co-resistance and multi-resistance between the two geographical regions were assessed by chi-square test. The associations of resistance, co-resistance and multi-resistance with household characteristics within the two geographical regions were determined by odds ratios (OR) with 95% confidence intervals. *P* < 0.05 was considered statistically significant. In the tables, the values of percentages are presented as integer numbers.

## 3. Results

### 3.1. Information on Households and Isolation Rate of *E. coli*

A total of 1251 samples of children’s stools, cow-dung and drinking water (417 of each sample) were collected from 417 households. Among these, 696 samples yielded positive cultures for E. coli. Table 2 summarizes household characteristics and E. coli recovery rates according to geographic location. 

**Table 2:** Households’ characteristics and *E. coli* isolation rate from various sources in non-coastal and coastal environment.

Information on households	NCE (*N* = 230), *n* (%)	CE (*N* = 187), *n* (%)
Socioeconomic status		
Lower	197 (86)	148 (79)
Upper	33 (14)	39 (21)
Education of family head		
Illiterate	37 (16)	18 (10)
Primary (1–5 years)	101 (44)	77 (41)
Secondary (6–12 years)	81 (35)	76 (41)
Higher (more than 12 years)	11 (5)	16 (8)
Drinking water sources		
Tube well	192 (83)	169 (90)
Water supply system	28 (12)	5 (3)
Well	4 (2)	13 (7)
Pond	6 (3)	0
Defecation		
Latrine	26 (11)	30 (16)
Open-air	204 (89)	157 (84)
Age of child		
3 to 5 years	105 (46)	74 (40)
6 to 9 years	125 (54)	113 (60)
Sex of child		
Boy	128 (56)	102 (55)
Girl	102 (44)	85 (45)
Antibiotic use in the child, last year		
Yes	175 (76)	175 (94)
No	3 (1)	6 (3)
Not known	52 (22)	6 (3)
Antibiotic use in the cow, last year		
Yes	11 (5)	7 (4)
No	219 (95)	180 (96)
Isolation rate of *E. coli*		
Children’s stools	139 (60)	138 (74)
Cow-dung	140 (61)	128 (68)
Drinking water	97 (42)	54 (29)

NCE = Non-coastal Environment; CE = Coastal Environment; *N* = Total number of samples; *n* = Samples with observed variable.

### 3.2. Antibiotic Resistance Pattern

In our study, 92%, 90% and 96% of *E. coli* isolates from children’s stool, cow-dung and drinking water respectively, was resistant to at least one of the tested antibiotics. A comparison of the antibiotic resistance pattern of *E. coli* isolates from children’s stools, cow-dung, and drinking water from non-coastal and coastal environment is presented in Table 3. *E. coli* isolates from the non-coastal region, regardless of type of sample, showed significantly higher resistance prevalence to both 2nd generation cephalosporins (cefuroxime) and 3rd generation cephalosporins (cefotaxime and cefixime) and nalidixic acid. Norfloxacin resistance was significantly higher in non-coastal cow-dung isolates. Ciprofloxacin resistance was significantly higher in non-coastal drinking water isolates only. Additionally, non-coastal cow-dung and drinking water *E. coli* isolates had significantly higher prevalence of resistance to tetracycline and ampicillin/sulbactam. Although not significant, the only exception from non-coastal isolates having higher resistance prevalence was the higher amikacin resistance among *E. coli* isolates from children’s stool in the coastal region.

Table 4 shows the co-resistance and multi-resistance of *E. coli* isolates from children’s stool, cow-dung and drinking water from the non-coastal and coastal environment to different groups of antibiotics. In general, we found that the co-resistance and multi-resistance in *E. coli* isolates was higher in the non-coastal as compared to the coastal region.

**Table 3:** Antibiotic resistance pattern of *E. coli* isolated from children’s stool, cow-dung, and drinking water originating from non-coastal and coastal environment.

Antibiotics	Resistance in *E. coli* isolates from various sources								
	Children’s stool, *n* (%)			Cow-dung, *n* (%)			Drinking water, *n* (%)		
	NCE *N* = 139	CE *N* = 138	*P*	NCE *N* = 140	CE *N* = 128	*P*	NCE *N* = 97	CE *N* = 54	*P*
Tetracycline	76 (55)	70 (51)	0.51	69 (49)	46 (36)	**0.027**	59 (61)	16 (30)	**<0.001**
Ampicillin/Sulbactam	69 (50)	55 (40)	0.102	71 (51)	35 (27)	**<0.001**	46 (47)	16 (30)	**0.033**
Cefuroxime (2nd)	88 (63)	70 (51)	**0.034**	89 (64)	48 (37)	**<0.001**	63 (65)	22 (41)	**0.004**
Cefotaxime (3rd)	90 (65)	68 (49)	**0.009**	82 (59)	39 (30)	**<0.001**	72 (74)	23 (43)	**<0.001**
Cefixime (3rd)	95 (68)	72 (52)	**0.006**	86 (61)	49 (38)	**<0.001**	52 (54)	16 (30)	**0.005**
Cotrimoxazole	79 (57)	52 (38)	**0.001**	69 (49)	37 (29)	**0.001**	51 (53)	22 (41)	0.163
Amikacin	39 (28)	52 (38)	0.088	58 (41)	42 (33)	0.145	25 (26)	9 (17)	0.199
Ciprofloxacin	60 (43)	56 (41)	0.663	43 (31)	33 (26)	0.371	40 (41)	11 (20)	**0.009**
Norfloxacin	70 (50)	59 (43)	0.204	71 (51)	43 (34)	**0.005**	47 (48)	18 (33)	0.072
Nalidixic acid	92 (66)	71 (51)	**0.013**	78 (56)	48 (37)	**0.003**	63 (65)	20 (37)	**0.001**

*N* = Total number of samples; *n* = Resistant isolates; NCE = Non-coastal Environment; CE = Coastal Environment; 2nd = 2nd generation cephalosporins; 3rd = 3rd generation cephalosporins.

**Table 4:** Co-resistance and multi-resistance of *E. coli* in children’s stool, cow-dung and drinking water from non-coastal and coastal environment.

Antibiotics	Penicillin (B)		Cephalosporin (C) (cefotaxime, cefixime)		Cotrimoxazole (D)		Aminoglycoside (E)		Fluoroquinolone (F)	
	NCE	CE	NCE	CE	NCE	CE	NCE	CE	NCE	CE
**4A. Prevalence of resistance in children’s stool *n* (%)**										
Tetracycline (A)	35 (25)	34 (25)	68 (49)	52 (38)	44 (32)	30 (22)	21 (15)	33 (24)	49 (35)	41 (28)
Penicillin (B)			60 (43)	50 (36)	49 (35) **	28 (20)	23 (17)	20 (14)	51 (37)	51 (37)
Cephalosporin (C)					70 (50) **	42 (30)	29 (21)	38 (28)	74 (53)	62 (45)
Cotrimoxazole (D)							25 (18)	18 (13)	58 (42) *	41 (30)
Aminoglycoside (E)									24 (17)	29 (21)
AB			32 (23)	31 (22)	21 (15)	18 (13)	9 (6)	11 (8)	26 (19)	30 (22)
ABC					21 (15)	17 (12)	9 (6)	11 (8)	25 (18)	28 (20)
ABCD							5 (4)	6 (4)	17 (12)	16 (12)
ABCDE									4 (3)	6 (4)
**4B. Prevalence of resistance in cow-dung *n* (%)**										
Tetracycline (A)	35 (25) **	16 (13)	58 (41) **	29 (23)	36 (26) *	17 (13)	28 (20)	21 (16)	44 (31) **	22 (17)
Penicillin (B)			64 (46) ***	26 (20)	50 (36) **	22 (17)	31 (22) **	12 (9)	58 (41) **	31 (24)
Cephalosporin (C)					61 (44) ***	29 (23)	43 (31)	26 (20)	73 (52) **	40 (31)
Cotrimoxazole (D)							30 (21)	17 (13)	53 (38) **	30 (23)
Aminoglycoside (E)									34 (24) **	17 (13)
AB			33 (24) *	15 (12)	22 (16)	11 (9)	14 (10)	6 (5)	28 (20) *	14 (11)
ABC					20 (14)	10 (8)	12 (9)	5 (4)	26 (19)	14 (11)
ABCD							6 (4)	5 (4)	14 (10)	10 (8)
ABCDE									3 (2)	5 (4)
**4C. Prevalence of resistance in drinking water *n* (%)**										
Tetracycline (A)	29 (30) *	8 (15)	56 (58) ***	13 (24)	31 (32) *	9 (17)	11 (11)	4 (7)	38 (39) **	9 (17)
Penicillin (B)			38 (39) *	11 (20)	37 (38) *	11 (20)	18 (19) **	1 (2)	34 (35) *	10 (19)
Cephalosporin (C)					40 (41)	16 (30)	17 (18)	4 (8)	55 (57) **	17 (31)
Cotrimoxazole (D)							17 (18) *	2 (4)	37 (38) *	11 (20)
Aminoglycoside (E)									17 (18) *	2 (4)
AB			28 (29)	8 (15)	22 (23) *	5 (9)	6 (6)	1 (2)	21 (22)	6 (11)
ABC					21 (22)	5 (9)	5 (5)	1 (2)	20 (21)	6 (11)
ABCD							2 (2)	0	16 (16)	4 (7)
ABCDE									2 (2)	0

*n* = number of isolates showing resistance; NCE = Non-coastal Environment; CE = Coastal Environment; Chi-square test: * *P* < 0.05, ** *P* < 0.01, *** *P* < 0.001.

### 3.3. Association of Resistance, Co-Resistance and Multi-Resistance

The association between the resistance patterns (co-resistance and multi-resistance) of *E. coli* isolates and the type of sample, geographic location and the households’ information was estimated. In *E. coli* isolates from children’s stools, resistance was associated with geographical region (OR = 3.1, 95% CI 1.18–8.01, *P* = 0.021) with higher prevalence in the non-coastal than coastal region. Furthermore, resistance was also associated with sex of the children. Boys had 2.9 times higher odds (95% CI 1.21–6.86, *P* = 0.016) to carry a resistant isolate than girls, irrespective of the region to which they belonged. Co-resistance and multi-resistance were not associated with any of the variables.Among *E. coli* strains isolated from cow-dung, resistance (OR = 3.6, 95% CI 1.59–8.03, *P* = 0.002), co-resistance (OR = 2.5, 95% CI 1.39–4.37, *P* = 0.002) and multi-resistance (OR = 2.2, 95% CI 1.12–4.34, *P* = 0.022) were associated with geographical region, with higher prevalence in non-coastal areas. Similarly, in *E. coli* isolates from drinking water, resistance (OR = 3.8, 95% CI 1.00–14.44, *P* = 0.049), co-resistance (OR = 3.2, 95% CI 1.36–7.41, P = 0.008) and multi-resistance (OR = 2.8, 95% CI 1.06–7.07, *P* = 0.036) were associated with geographical region and the prevalence was higher in the non-coastal region. The association between resistance pattern (resistance, co-resistance and multi-resistance) in *E. coli* isolates and household’s information within each geographical region was also estimated. Within the non-coastal region stratum the multi-resistance (OR 3.2, 95% CI 1.21–8.51, *P* = 0.019) in *E. coli* isolates from children’s stool were higher among boys than the girls. 

Apart from the above, none of the compared variables were associated with resistance, co-resistance or multi-resistance in *E. coli* isolates. 

### 3.4. Ciprofloxacin MIC Determination

**Figure 1:**
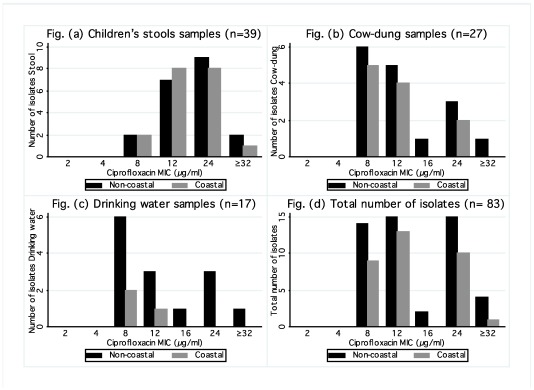
Ciprofloxacin MIC (Minimum inhibitory concentration) values among ciprofloxacin-resistant *E. coli* isolates from (**a**) Children’s stools samples (*n* = 39); (**b**) Cow-dung samples (*n* = 27); (**c**) Drinking water samples (*n* = 17); and (**d**) Total number of isolates (*n* = 83).

Ciprofloxacin MIC values ranged from 8 to 32 μg/mL, regardless of type of sample and geographic location, as shown in Figure 1. *E. coli* isolates from children’s stools had significantly higher levels of resistance (≥24 µg/mL, *P* = 0.025) when compared to cow-dung and drinking water isolates. Amongst cow-dung *E. coli* isolates, the highest level of resistance (≥32 µg/mL) was observed in isolates from the non-coastal region only. Overall, *E. coli* isolates originating from the non-coastal region tended to have higher levels of resistance as compared to those from the coastal region, albeit not statistically significant.

## 4. Discussion

In our study we compared antibiotic resistance in two regions with distinct geographical and environmental attributes. To our knowledge, this is the first study investigating the association of antibiotic resistance patterns in *E. coli* isolates from community and environmental sources like children’s stools, cow-dung and drinking water with two different natural environments and their attributes. We identified a higher prevalence of resistance in isolates from all the sources in the non-coastal area compared to the coastal area. Compared to the coastal region, the distinguishing contrasting physical and natural environmental features of the non-coastal region were—maximum temperature 11 °C higher, height from sea-level 190 meters more, forest cover about 12 times more, population density 4.6 times less and literacy rate nearly half. As there is a lack of studies similar to ours it is difficult to say whether the combined influence of these environmental features has any contribution towards our results. In addition, the social environmental characteristics of these regions could also have contributed to the results. 

High prevalence of antibiotic resistance (92% to at least one of the tested antibiotics and 24% multi-resistance) among normal faecal flora *E. coli* isolates from children was observed in our study. In contrast, a previous study from Tamil Nadu, India carried out in 2005 found a prevalence 63% resistance to at least one antibiotic and 32% multi-resistance in *E. coli* in healthy children’s stools [19] and a study in Greece conducted in 1998 reported that 40% of healthy children carried resistant *E. coli* [20]. A community-based study among young children in Peru suggested that environmental contamination with resistant bacteria significantly contribute to children’s carriage of antibiotic-resistant *E. coli* [21]. Our study was conducted in villages in India, where sanitation generally is very poor and hence such a possibility cannot be ruled out in our study. Furthermore, we found that the prevalence of resistance in *E. coli* isolated from children’s stool was higher among boys than girls in the non-coastal region. Somewhat contradictory data are available from other contexts. For example, a community study from United Kingdom on susceptibility in urinary coliform isolates found that resistance was more frequent in isolates from boys [22], whereas, a study from Greece found that resistance in commensal *E. coli* isolates was higher when they were from girls [20]. 

Veterinary use of antibiotics has been shown to result in antibiotics resistance in commensal *E. coli* isolates from farm cattle faeces [23]. A study in Pennsylvania, United States found 40% multidrug-resistance among *E. coli* isolates in cow-dung from healthy lactating cows (no information was available on individual antibiotic treatment) [24]. In our study 19% multi-resistance *E. coli* isolates were observed in cow-dung, although participants informed that only 5% of cows had been given antibiotic treatment. Antibiotics and antibiotic resistant bacteria in soil and aquatic environment [25] and antibiotic resistant *E. coli* from human and animal sources [26] have been previously documented. The selection of resistant bacteria at very low antibiotic concentrations have also been reported [27]. A previous study found that faecal pollution in rural water and rural watershed was dominated by cattle [28] as the source of resistance bacteria. More than 80% of households in our study did not have latrines but followed open-air defecation practice, which could also be one of the pathways for spread of antibiotic residuals and resistant bacteria in the grassland and aquatic environments [25]. In the area of our study, cows generally graze in open fields and besides household water sources, drink water from open water sources like ponds, rivulets, stagnant water *etc*. It is possible that this might have had a role to play in the development of resistance of *E. coli* detected in cow-dung.

We found that 24% of *E. coli* isolated from drinking water was multi-resistant. A study in Hyderabad, Pakistan found 63% multi-resistance *E. coli* isolates from drinking water [29] and a study from Tamil Nadu, India found eight of nine drinking water samples were resistant to at least one tested antibiotic [19]. The importance of emerging pathogens in drinking water has been documented globally [30]. Antibiotic resistant bacteria have been found in Indian aquatic environment e.g., hospital waste water [31]. 

Some comparative antibiotic resistance studies of different nature are found in the literature. Regional differences in antibiotic resistance have previously been documented [32,33] and it is suggested that this could result from different local co-selective events like antibiotic pressure and independent clonal spread in each region [33]. For example, *E. coli* isolates from point sources (industrial and municipal effluents) have been shown to have higher resistance compared to nonpoint sources (land runoff and septic tank seepage) [34]. Previous studies from Tamil Nadu, India [19] and from South Africa [35] have shown that prevalence of antibiotic resistance among commensal bacteria was slightly higher in rural population compared to urban population. However a study among adults from eight developing countries found the contrasting result that the prevalence of resistance in faecal *E. coli* was more common in urban than in rural areas [36].

In our previous qualitative study [37] in the same settings the healthcare professionals perceived that behavioural and social environmental factors along with biophysical environmental factors could contribute to resistance development in different geographical settings. It was also found that in the non-coastal region of “Malkangiri”, there is a lack of registered allopathic doctors, hence so called “quacks” (persons with no medical qualification) and some of the homeopathic and ayurvedic (Indian system of medicine) healthcare providers commonly prescribe antibiotic treatment (although they are not legally authorised to prescribe antibiotics). Compared to this, in the coastal region of “Puri” healthcare facilities are better and there is availability of higher numbers of trained allopathic doctors [37]. In both cases unnecessary or irrational antibiotic prescribing might be present. However, the trained healthcare providers could be expected to have comparatively more rational prescribing compared to the untrained prescribers. Additionally, in the present study antibiotic use among children in the non-coastal areas might have been underestimated since 22% of the respondents did not know if their child had taken antibiotics in the last year, while the corresponding figure was only 3% in coastal areas. Irrational/unnecessary antibiotic treatment has been cited as a cause of resistance development [4,9]. Surprisingly, a previous study in tropical South America found that heavy use of chloroquine to treat malaria likely selected for ciprofloxacin resistance in *E. coli* [38]. This might be one reason for the relatively higher quinolone resistance in Malkangiri (non-coastal) as chloroquine has been a commonly used medicine due to endemic malaria. 

The impact of antibiotics or resistant bacteria on humans might happen in several ways. When contaminated food is ingested, the bacteria might transfer resistance determinants to other bacteria in the human gut, so called horizontal gene transfer [7,8,39]. Additionally, antibiotic residues in food products or water may allow the selection of antibiotic-resistant bacteria after the food or water is consumed [10]. Through excretion resistant bacteria might flow from the gut of humans and animals into soil/terrain and water. Therefore, there might be interaction between *E. coli* from the natural environment (soil/terrain and water), humans and animals. 

The main strength of this study is that it has isolated and compared *E. coli* obtained from three different sources (children’s stool, cow-dung and drinking water) from the same household in two different contrasting natural environments. Furthermore, it has determined the antibacterial susceptibility pattern using standardised methods for the obtained isolates. In addition, MIC determination was performed for a sub-set of ciprofloxacin-resistant isolates. However, there are some limitations in this study. In spite of collecting about 34% excess isolates in case of children’s stool, still the calculated number of isolates from drinking water could not be reached. Nevertheless, there were still significant differences in *E. coli* resistance prevalence to most of the antibiotics tested between the two geographical regions. However, it cannot be ruled out that a larger sample size would have led to significantly different results between the two regions also for the remaining antibiotics. Another limitation was that we tested only one isolate per sample. However, in our pilot study we tested five isolates per sample and obtained almost identical resistance patterns for all isolates from the same sample. Thus, as we had limited funding, we decided to only include one isolate from each sample in the main study. 

## 5. Conclusions

In conclusion, this study shows that the overall prevalence of antibiotic resistance in *E. coli* isolated from children’s stool, cow-dung and drinking water was high and even higher in the non-coastal than the coastal environment. Our findings suggest the need for better understanding of the interaction between human and animal faecal bacteria and the environment. Furthermore, this study implies the need for better drinking water quality management in rural Odisha, India. In addition, these findings can be used for improving antibiotic management. 
